# Protective effects of lavender oil on sepsis-induced acute lung injury via regulation of the NF-κB pathway

**DOI:** 10.1080/13880209.2022.2067570

**Published:** 2022-05-19

**Authors:** Qian Xie, Yi Wang, Guo-Liang Zou

**Affiliations:** a Pediatrics Department, Heilongjiang Provincial Hospital, Harbin, Heilongjiang, China; b Pediatrics Department, The Sanya Hongsen Hospital of Harbin Medical Universit, Sanya, Hainan, China; c No.1 Cardiovascular Disease Department, Heilongjiang Chinese Medical University No.1 Affiliated Hospital, Harbin, Heilongjiang, China

**Keywords:** Caecal ligation and puncture, inflammation, oxidative stress, apoptosis

## Abstract

**Context:**

Lavender oil (Lav) from *Lavandula angustifolia* L. (Lamiacease) exhibits antioxidative and anti-inflammatory properties against various diseases.

**Objective:**

The study explores the effect of Lav pre-treatment on sepsis-induced acute lung injury (ALI).

**Materials and methods:**

Sprague-Dawley rats were assigned into Sham, caecal ligation and puncture (CLP), CLP + Lav (200, 400, and 800 mg/kg) groups. Lav was administered by gavage, once a day, for 7 days. Histological analysis was performed using haematoxylin and eosin staining. Cytokine and nitrite levels were detected by enzyme-linked immunosorbent assay kits and Griess reagent. Gene and protein expression were tested by quantitative real-time polymerase chain reaction and western blot.

**Results:**

The levels of tumour necrosis factor-α (BALF: 64%, serum: 59%), interleukin (IL)-1β (BALF: 63%, serum: 66%) and IL-6 (BALF: 54%, serum: 59%), and nitrite (40%) and inducible nitric oxide synthase (51%), and the level of myeloperoxidase (66%) and malondialdehyde (59%), and cleaved-caspase 3 (84%) and Bax expression (74%) induced by CLP were decreased when given Lav. Additionally, the level of superoxide dismutase (211%) and glutathione (139%), and the expression of Bcl-2 (980%) induced by CLP were increased when given Lav. The increased p-nuclear factor (NF)-κB/NF-κB (72%) and p-inhibitor of κBα (IκBα)/IκBα (77%) induced by CLP could be reversed by Lav.

**Discussion and conclusions:**

Lav pre-treatment might protect rats from sepsis-induced ALI via deactivation of the NF-κB pathway. Our research demonstrated the regulatory mechanisms of Lav in sepsis-induced ALI and can provide a theoretical basis for the use of Lav in the treatment of sepsis-induced ALI.

## Introduction

Sepsis is a systemic inflammatory response syndrome caused by severe infection with high mortality rates (Taşcı et al. 2017). Researchers have discovered that sepsis is usually accompanied by multiple organ dysfunction, and organ failure is a major cause of sepsis-related death. Among multiple organs, the lungs are the most vulnerable in the process of sepsis, and nearly half the patients with severe sepsis develop acute lung injury (ALI) (Chen et al. 2017; Park et al. 2019). ALI has been recognised as a primary reason for sepsis-related mortality owing to its early occurrence and high incidence rate (Bastarache et al. 2006). ALI is characterised by pulmonary edema, neutrophil infiltration, alveolar or interstitial thickening and protein-rich fluid accumulation in the airspaces (Matute-Bello et al. 2008). Oxidative damage marked by nitration and lipid peroxidation and the resulting acute inflammatory response are essential pathological processes in ALI (Vadász et al. 2005; Yang et al. 2019). In recent years, several studies have explored the functions of multiple synthetic or natural chemicals, such as hypaphorine, notoginsenoside R1, and butorphanol, in impairing the inflammatory responses in sepsis-induced ALI (Cao et al. 2021; Ding et al. 2021; Luan et al. 2021). However, the clinical application of these findings remains limited. Hence, it is still necessary to identify novel chemicals that are effective for the future treatment of sepsis-induced ALI.

Lavender, a member of the Lamiacease family, is widely used in a variety of herbal treatments and cosmetic products (Vakili et al. 2014; Souri et al. 2019). Lavender oil (Lav), extracted from the flower of *Lavandula angustifolia* L., has been reported to have anti-inflammatory, antioxidative, antinociceptive, antimicrobial, anti-depressive and anticancer properties in various investigations (Panahi et al. 2014; Silva et al. 2015; Qadeer et al. 2018; Souri et al. 2019; Boukhatem et al. 2020). For instance, lavender essential oil (LVO) combined with meropenem treatment-induced oxidative stress in *Klebsiella pneumoniae* carbapenemase-producing *K. pneumoniae* (KPC-KP) cells by elevating the levels of lipid peroxidation and ROS, thereby repressing the growth of KPC-KP, ultimately reducing the threat to human health (Yang et al. 2020). Another study revealed that Lav alleviated renal ischemia/reperfusion injury in rats by repressing oxidative stress, inflammation and cell apoptosis (Aboutaleb et al. 2019). Based on these findings and the pathological characteristics of ALI, we speculated that Lav may also play a role in the treatment of ALI.

In this research, a sepsis animal model was constructed by performing caecal ligation and puncture (CLP) with different doses of Lav pre-treatment carried out on the rats. Various factors, including histological and morphological changes in lung tissues, total protein content and inflammatory cell number in bronchoalveolar lavage fluid (BALF), were determined to confirm whether the ALI model was successfully created. After that, the levels of inflammatory cytokines and nitrification-, oxidative stress- and apoptosis-related factors were detected to explore the influence of Lav pre-treatment on ALI of septic rats. Additionally, the bioinformatic analysis combined with western blot and immunohistochemistry was conducted to determine the roles of the nuclear factor (NF)-κB pathway in sepsis-induced ALI. This study revealed the regulatory mechanisms of Lav in sepsis-induced ALI, which may provide a theoretical basis for the use of Lav in the treatment of ALI.

## Materials and methods

### Animal treatments

A total of 30 male Sprague-Dawley (SD) rats (aged 8–10 weeks and weighing 220–250 g) were obtained from Beijing Vital River Bioscience Co., Ltd. (Beijing, China). All rats were housed under appropriately controlled environmental conditions (humidity, 60–65%; temperature, 22–25 °C, 12 h light/dark cycles, and free access to standard food and water) for 7 days to adapt to the laboratory environment. This study was approved by the Ethics Committee of the Heilongjiang Provincial Hospital (no. HLJDW20200602). Then, the rats were randomly assigned into 5 groups, including the Sham, CLP, CLP + Lav (200 mg/kg), CLP + Lav (400 mg/kg) and CLP + Lav (800 mg/kg) groups, with 6 rats in each group. The doses of Lav were determined based on previous studies (Silva et al. 2015; Sadeghzadeh et al. 2017; Aboutaleb et al. 2019).

Lav was purchased from Aromarant Co. Ltd. (Rottingen, Germany). In this study, Lav was first dissolved in dimethyl sulfoxide (DMSO) to prepare a stock solution, and then diluted to the indicated concentrations. After fasting for 8 h, intragastric administration of 4 mL of Lav was performed in each group with the help of a No.16 needle. The administration was conducted once a day for 7 consecutive days. After the last administration, the rats have fasted for 12 h, and CLP was performed.

### Construction of sepsis-induced ALI model in rats

A sepsis-induced ALI model was constructed using CLP as described previously (Zhang et al. 2020). Briefly, the rats were anaesthetised by intraperitoneal injection of sodium phenobarbital (50 mg/kg). After that, an incision was created in the middle of the abdomen, and the caecum was exposed, ligated at 1/2 and punctured with a No.18 needle at the ligation end. A small number of faeces was extruded from the pinhole. Finally, the caecum was returned, and the incision was sutured, immediately followed by a subcutaneous injection of 30 mL/kg of normal saline solution. In the Sham group, the caecum was also exposed, but without ligation and perforation.

### Collection of blood from sepsis-induced ALI model

The rats were anaesthetised with sodium pentobarbital (200 mg/kg, intraperitoneal) 24 h after the operation. Subsequently, blood samples were collected from the abdominal aorta in the presence of heparin sodium (Solarbio, Beijing, China). The serum was obtained after centrifugation and stored at −20 °C for follow-up experiments.

### BALF collection, cell counting and protein concentration determination

After the rats were anaesthetised with sodium pentobarbital (200 mg/kg, intraperitoneal), the BALF was collected. The preparations of BALF collection consisted of incising the skin of the larynx to expose the trachea, inserting the catheter into the trachea from the mouth, and tying the trachea and catheter tightly using a surgical thread. After that, 1 mL of sterile phosphate-buffered saline (PBS) was injected into the lungs via the tracheal cannula and BALF was subsequently collected. This procedure was repeated 3 times. The collected BALF was centrifuged at 1200 × *g* at 4 °C for 10 min, and the supernatant was collected, used for protein concentration determination with a BCA protein assay kit (Solarbio), and stored at −20 °C for further cytokine detection. The obtained cell pellets were then resuspended in PBS to determine the number of whole cells and neutrophils using a blood counting chamber (Solarbio) and Wright-Giemsa staining methods, respectively.

### Lung tissues collection and exudation detection (wet/dry weight ratio determination)

Following this procedure, the rats were euthanized with sodium pentobarbital (200 mg/kg, intraperitoneal). Lung tissue was removed, and the right lung tissue of each group was immediately rinsed with PBS, dried with absorbent paper, weighed and recorded as wet weight. These tissues were then dehydrated in an incubator at 80 °C for 48 h until the weight remained constant, weighed again, and recorded as the dry weight. Finally, the wet/dry weight ratio of lung tissue was calculated. Other parts of the lung tissue were immediately frozen in liquid nitrogen or fixed in 4% paraformaldehyde (PFA) for follow-up experiments.

### Histopathological analysis and lung injury score

The left lung tissues were fixed with 4% PFA for 72 h, dehydrated with gradient ethanol, and embedded in paraffin. The tissues were sliced into 4 μm sections, de-paraffinized, hydrated, stained with haematoxylin and eosin (H&E), and treated with gradient alcohol and xylene. Finally, the slides were sealed with neutral gum (Solarbio). Histopathological features, including alveolar cavity edema, alveolar or interstitial thickening, and inflammatory cell infiltration, were determined using a light microscope (MOTIC, China). Histological scores were determined based on the recorded histopathological features. The score range was 0–4, with a higher number indicating the increasing severity of the disease.

### Determination of cytokine levels in the serum and BALF

The levels of cytokines, including tumour necrosis factor-α (TNF-α), interleukin (IL)-1β and IL-6, in the serum and BALF were tested using the following enzyme-linked immunosorbent assay (ELISA) kits: Rat TNF-α ELISA kit (ab100785, Abcam, Cambridge, UK), Rat IL-1β ELISA kit (ab100767, Abcam) and Rat IL-6 ELISA kit (ab100772, Abcam). All experiments were performed in accordance with the product instructions.

### Measurement of nitrite content in lung tissues

The collected lung tissues were homogenised in PBS. The homogenates were centrifuged at 5000 rpm for 15 min and the supernatant was collected. After that, nitrite determination was using the Griess reagent kit (Thermo Fisher Scientific, MA, USA) following the manufacturer’s instructions.

### Determination of inducible nitric oxide synthase (iNOS) level in lung tissues

The mRNA level of iNOS in lung tissue was determined by quantitative real-time polymerase chain reaction (qRT-PCR) as described previously (Rungsung et al. 2018). In brief, total RNAs were isolated from the collected lung tissues with the help of Trizol reagent (TAKARA, Beijing, China) and were finally dissolved in diethylpyrocarbonate (DEPC; MACKLIN, Shanghai, China)-treated ddH_2_O. Afterwards, the first strand of cDNA synthesis was fulfilled using a BeyoRT™ III First Strand cDNA Synthesis Kit (Beyotime Biotechnology, Shanghai, China). Then, the iNOS expression was determined using BeyoFast™ SYBR Green One-Step qRT-PCR Kit (Beyotime Biotechnology) with a Stratagene Mx3000P instrument (Agilent Technologies, California, USA). GAPDH was used as the internal control, and the 2^-ΔΔCt^ method was used to calculate iNOS expression. The sequences were as follows: inducible nitric oxide synthase (iNOS) (Forward) 5′-GGTGCTATTCCCAGCCCAA-3′, (Reverse) 5′-AGTCACATGCAGCTTGTCCA-3′; GAPDH (Forward) 5′-AGACAGCCGCATCTTCTTGT-3′, (Reverse) 5′-CCGATACGGCCAAATCCGTT-3′.

### Measurements of iNOS, apoptosis- and NF-κB pathway-associated factors expression in lung tissues

The protein levels of iNOS, apoptosis- and NF-κB pathway-associated factors were determined by western blot as described previously (Yuan et al. 2019). Total cellular protein was extracted using a radioimmunoprecipitation assay (RIPA) lysis buffer (Beyotime Biotechnology). Then, protein concentration was quantified using a Nanodrop 2000 system (Thermo Fisher Scientific). Protein separation was conducted using sodium dodecyl sulfate-polyacrylamide gel electrophoresis (SDS-PAGE), and the separated proteins were transferred onto nitrocellulose membranes. After blocking in 5% BSA (Beyotime Biotechnology) for 1 h, the primary antibodies were incubated at 4 °C overnight. Thereafter, the membranes were incubated with horseradish peroxidase-conjugated secondary antibody (ab205718, Abcam) at 25 °C for 1 h. Finally, the signals of the indicated proteins were visualised using an enhanced chemiluminescence system (Thermo Fisher, CA, USA).

The primary antibodies used in this experiment are detailed below: anti-iNOS (ab283655, Abcam), anti-cleaved-caspase 3 (#9661, Cell Signalling Technology, MA, USA), anti-Bcl-2 (ab196495, Abcam), anti-Bax (ab182734, Abcam), anti-p-NF-κB (#3031, Cell Signalling Technology), anti-NF-κB (#8242, Cell Signalling Technology), anti-inhibitor of κBα (IκBα) (#9242, Cell Signalling Technology), anti-p-IκBα (#2859, Cell Signalling Technology), anti-β-actin (#4967, Cell Signalling Technology).

### Measurements of myeloperoxidase (MPO) activity and malondialdehyde (MDA), superoxide dismutase (SOD) and glutathione (GSH) levels in lung tissues

MPO activity analysis and MDA, SOD and GSH level determination were, respectively, completed with the kit listed below: MPO (#A044-1-1), MDA (#A003-1), SOD (#A001-1) and GSH (#A006-2-1), were all provided by the Nanjing Jiancheng Biological Engineering Institute (Nanjing, China). All procedures were performed in accordance with the manufacturer’s instructions.

### Cell apoptosis determination

Cell apoptosis was determined using the TdT-mediated dUTP Nick-End Labelling (TUNEL) apoptosis assay kit (#C1091; Beyotime Biotechnology) as previously described (Zhu et al. 2019). Briefly, the embedded lung tissues were sliced into 4 μm sections. Then, these slices were deparaffinized with xylene, rehydrated with gradient ethanol and maintained with 20 μg/mL DNase-free Proteinase K at 30 °C for 30 min. After rinsing with PBS, the enhanced endogenous peroxidase blocking buffer (#P0100B, Beyotime Biotechnology) was incubated at 25 °C for 20 min. Then, the slices were maintained with a biotin labelling solution at 37 °C for 60 min under dark conditions and incubated with Streptavidin-HRP working solution at 25 °C for 30 min. Thereafter, the colour was developed by incubating the sections with diaminobenzidine (DAB) solution at 25 °C for 10 min, and the nuclei were stained with haematoxylin (#C0107, Beyotime Biotechnology). After dehydration and transparency, the slices were sealed with neutral gum (Solarbio) and observed under a light microscope (MOTIC, China).

### Bioinformatic analysis

To explore the signalling pathways involved in the regulation of Lav in sepsis-induced ALI we performed a Kyoto Encyclopaedia of Genes and Genomes (KEGG) pathway analysis of Lav, lung injury and pneumonia. Venn diagram analysis was carried out to identify overlapping signalling pathways. R (version 4.0.2) was used to create the bubble chart.

### Immunohistochemical (IHC) analysis

The collected lung tissues were fixed in 4% PFA, dehydrated in gradient ethanol, embedded in paraffin and sliced into 4 μm sections. Afterwards, the sections were deparaffinized in xylene, rehydrated with gradient ethanol and heated in citrate buffer for 30 min to induce antigen retrieval. Thereafter, the sections were incubated overnight with an anti-p-NF-κB (#3031, Cell Signalling Technology) antibody at 4 °C, then incubated with an HRP-conjugated goat anti-rabbit antibody (ab205718, Abcam) at 25 °C for 1 h, and stained with DAB reagent for 3 min. After that, haematoxylin counterstaining was performed, and the results were observed under a microscope.

### Statistical analysis

Each experiment was performed in triplicate. Data were analysed using GraphPad Prism software 8 (CA, USA) and presented as the mean ± SEM. Comparisons between two groups and among multiple groups were performed using Student’s *t*-test and one-way analysis of variance (ANOVA) followed by Tukey's *post hoc* test, respectively. *p* < 0.05 represents statistically significant.

## Results

### Lav pre-treatment relieved sepsis-induced ALI in rats

**Figure 1.**
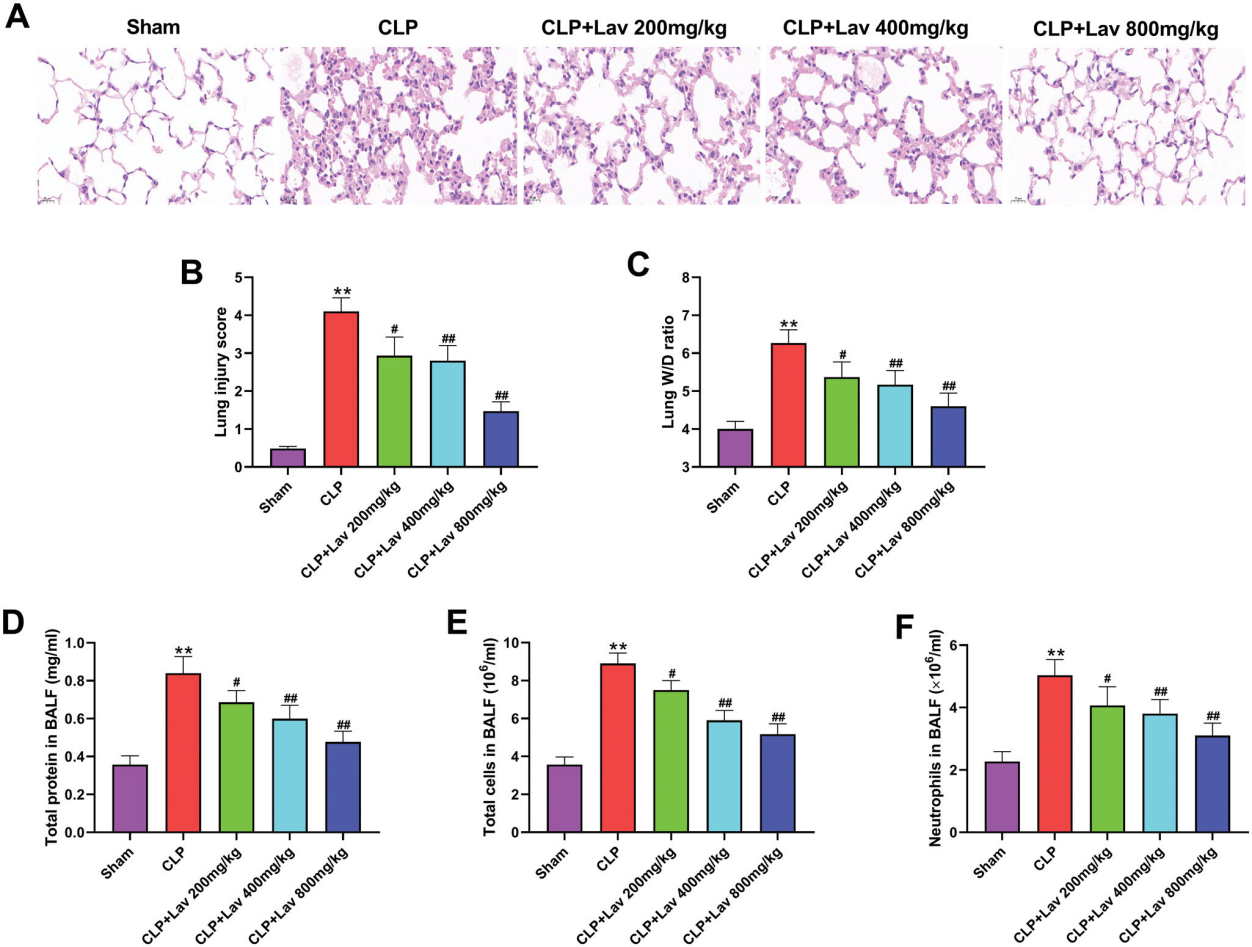
Lav pre-treatment relieved sepsis-induced ALI in rats. (A and B) The histological changes of the collected lung tissues were tested by H&E staining and the lung injury was scored. (C) The wet/dry ratio of the right lung was determined. (D–F) Total protein content, total cell and neutrophil numbers in BALF were tested via BCA assay kit, blood counting chamber and Giemsa staining, respectively. *Represents comparation with the Sham group, while # represents comparation with the CLP group. **p < 0.01, ^#^*p* < 0.05, ^##^*p* < 0.01.

In this research, a rat model of sepsis was successfully established by performing CLP surgery. Histopathological alterations were evaluated by H&E staining. The results showed that, compared with the Sham group, CLP resulted in alveolar cavity edema, alveolar or interstitial thickening and inflammatory cell infiltration (Figure 1A), which led to higher lung injury scores (Figure 1B, P < 0.01) and wet/dry ratio (Figure 1C, *P* < 0.01). Besides, the protein concentration and the number of total cells and neutrophils in BALF were measured to evaluate the therapeutic effects of Lav on sepsis-induced ALI. The results showed that compared with the Sham group, these factors were all distinctly augmented by CLP (Figure 1D–F, all *p* < 0.01). However, all the adverse effects induced by CLP were notably relieved by Lav pre-treatment (Figure 1A–F, *p* < 0.05 or *p* < 0.01). A combination of these results implies that Lav pre-treatment has protective effects on sepsis-induced ALI.

### Lav pre-treatment repressed inflammatory responses in sepsis-induced rats

**Figure 2.**
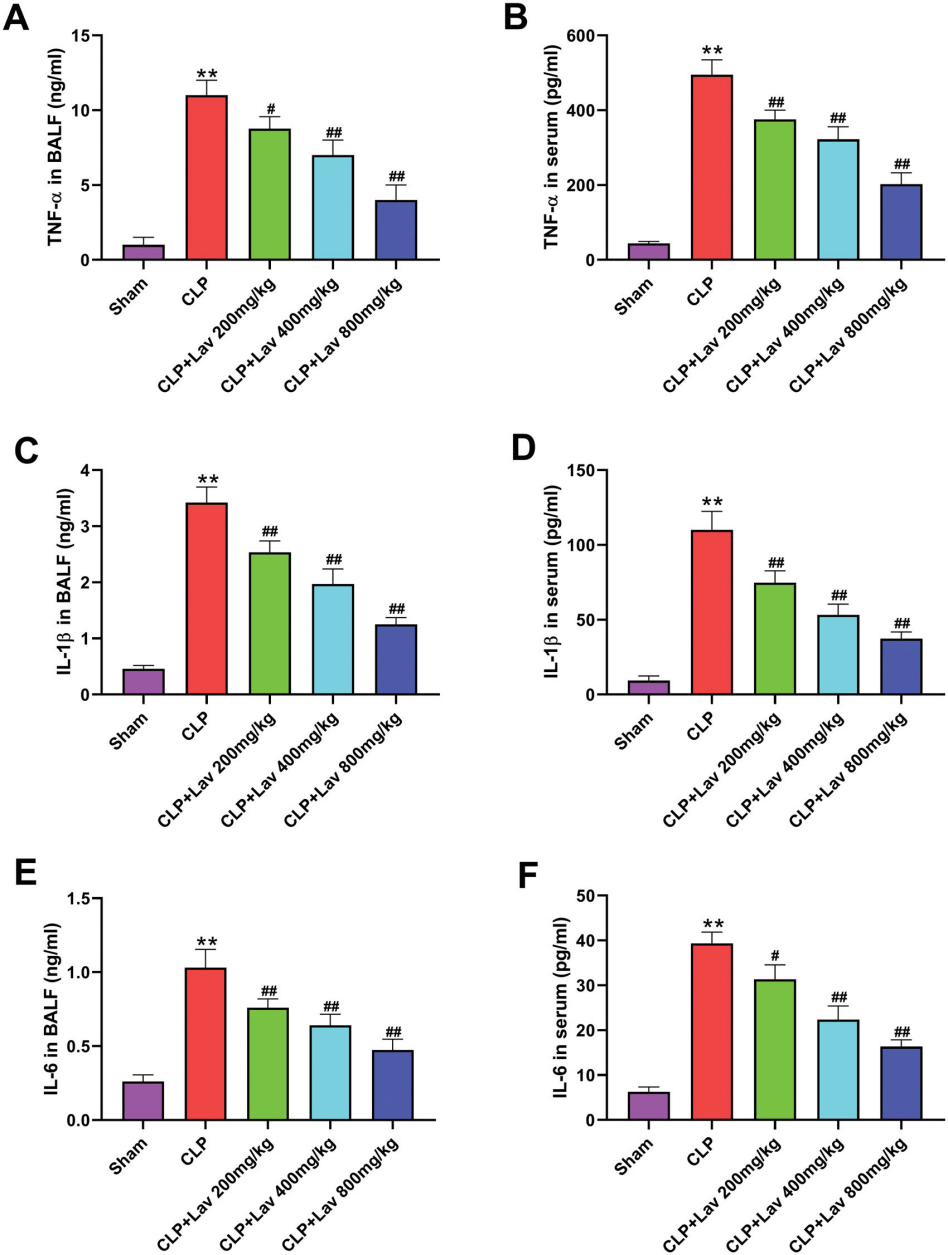
Lav pre-treatment repressed inflammatory responses in sepsis-induced rats. (A–F) The levels of inflammatory cytokines (IL-1β, IL-6 and TNF-α) in the BALF and serum were tested via the appropriate ELISA kits. *Represents comparation with the Sham group, while # represents comparation with the CLP group. **p < 0.01, ^#^*p* < 0.05, ^##^*p* < 0.01.

To explore the anti-inflammatory efficacy of Lav in sepsis-induced rats, the levels of TNF-α, IL-1β and IL-6 in the serum and BALF were tested by ELISA. The results showed that compared with the Sham group, the levels of TNF-α, IL-1β and IL-6 in both the serum and BALF were all distinctly elevated by CLP (*p* < 0.01), which were markedly mitigated by Lav pre-treatment (Figure 2A–F, *p* < 0.05 or *p* < 0.01). These results indicate that Lav pre-treatment could effectively restrain the inflammatory responses in sepsis-induced rats.

### Lav pre-treatment attenuates sepsis-induced nitrosative stress in the lung tissues of rats

**Figure 3.**
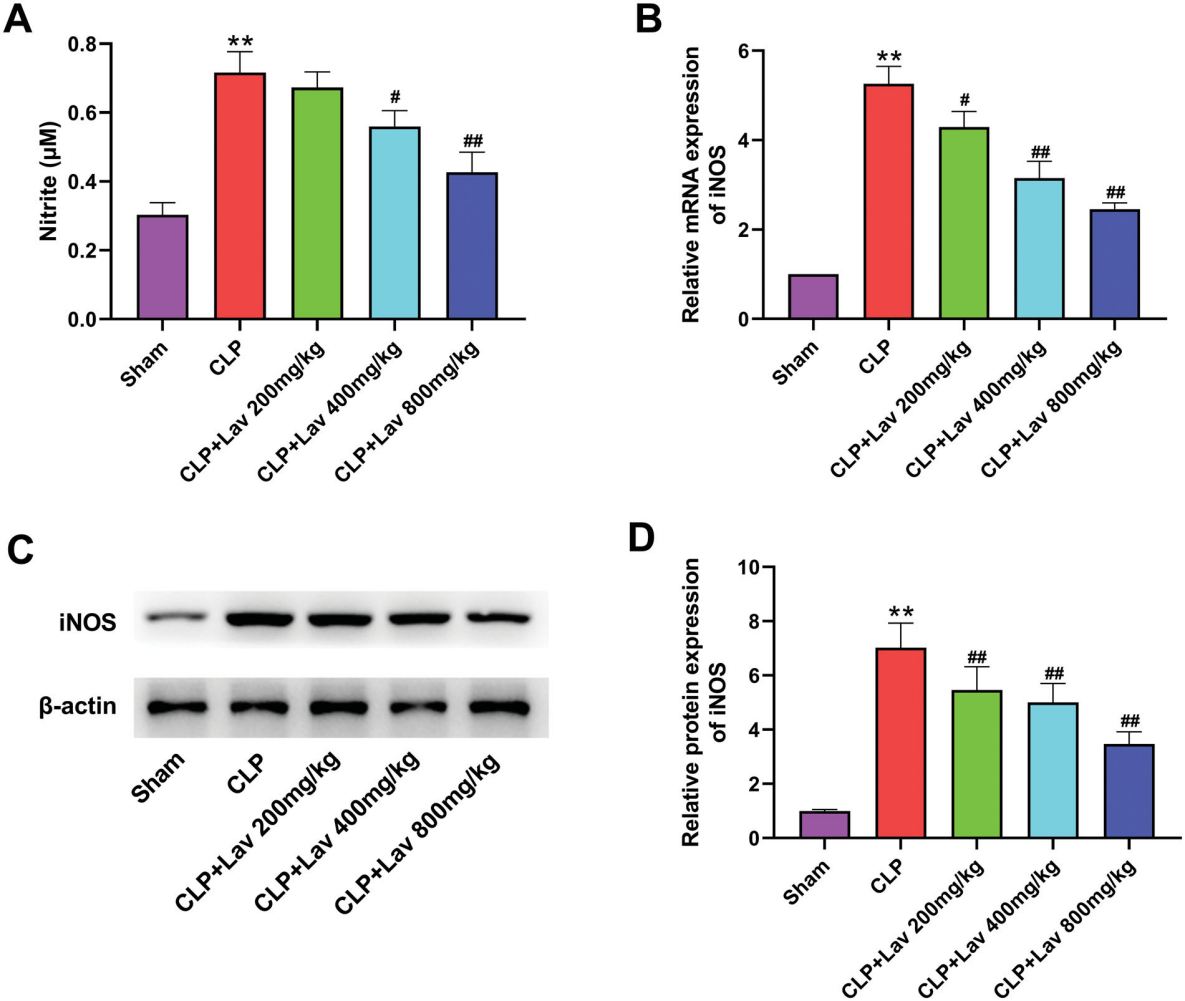
Lav pre-treatment attenuates sepsis-induced nitrosative stress in the lung tissues of rats. (A) The concentration of nitrite in the lung tissues was tested via Griess reagent. (B–D) The mRNA and protein levels of iNOS in the lung tissues were confirmed via qRT-PCR and western blot, respectively. *Represents comparation with the Sham group, while # represents comparation with the CLP group. **p < 0.01, ^#^*p* < 0.05, ^##^*p* < 0.01.

To confirm the efficacy of Lav pre-treatment on sepsis-induced nitrosative stress in rats, we examined nitrite levels and the mRNA and protein expression of iNOS in the collected lung tissues. Results demonstrated that the nitrite level, as well as the mRNA and protein levels of iNOS, were all distinctly elevated by CLP compared with the Sham group (Figure 3A–D, all *p* < 0.01). However, these elevating effects were all notably relieved by Lav pre-treatment (Figure 3A–D, *p* < 0.05 or *p* < 0.01). These results indicate that Lav pre-treatment could effectively protect lung tissues from CLP-induced nitrosative stress in rats.

### Lav pre-treatment alleviated sepsis-induced oxidative stress in the lung tissues of rats

**Figure 4.**
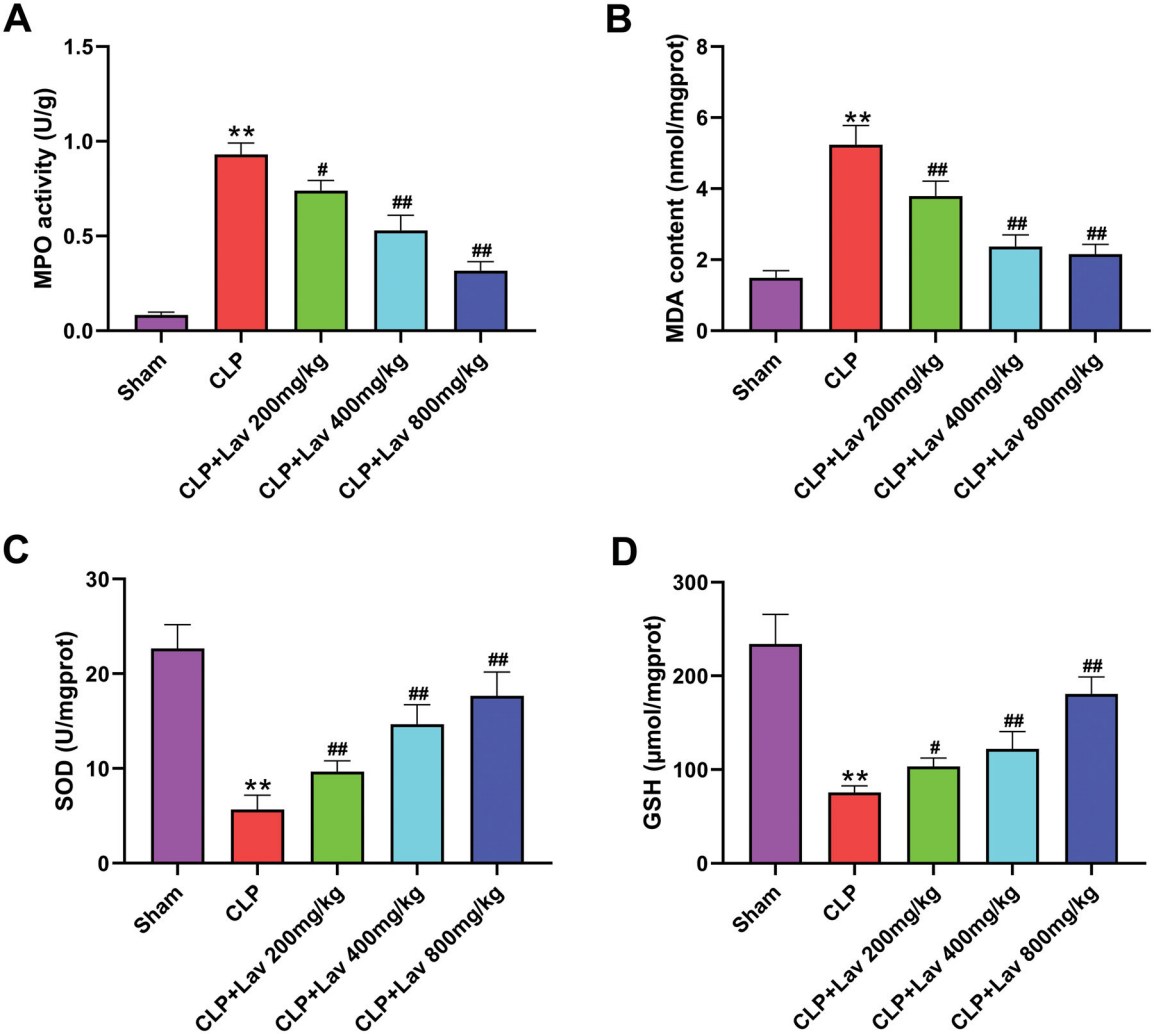
Lav pre-treatment alleviated sepsis-induced oxidative stress in the lung tissues of rats. (A) The MPO activity in the lung tissues was measured via a myeloperoxidase assay kit. (B–D) The contents of MDA, SOD and GSH in the lung tissues were tested with their corresponding kit. *Represents comparation with the Sham group, while # represents comparation with the CLP group. **p < 0.01, ^#^*p* < 0.05, ^##^*p* < 0.01.

To evaluate the effects of Lav pre-treatment on sepsis-induced oxidative stress in rats, we determined the levels of the oxidative stress injury indicators MPO and MDA, as well as the antioxidant stress injury indicators SOD and GSH in the collected lung tissues. Results displayed a significant increase in MPO activity and MDA content, and a notable reduction in SOD and GSH content in the CLP group compared to the Sham group (Figure 4A and B, all *p* < 0.01). Following results verified that Lav pre-treatment markedly reversed CLP-triggered effects, exhibiting that MPO activity and MDA content noticeably decreased with Lav pre-treatment, while SOD and GSH contents were observably augmented (Figure 4A–D, *p* < 0.05 or *p* < 0.01). Collectively, these data illustrate that Lav pre-treatment could protect rats against sepsis-induced oxidative stress.

### Lav pre-treatment protected the lung tissues from sepsis-induced cell apoptosis

**Figure 5.**
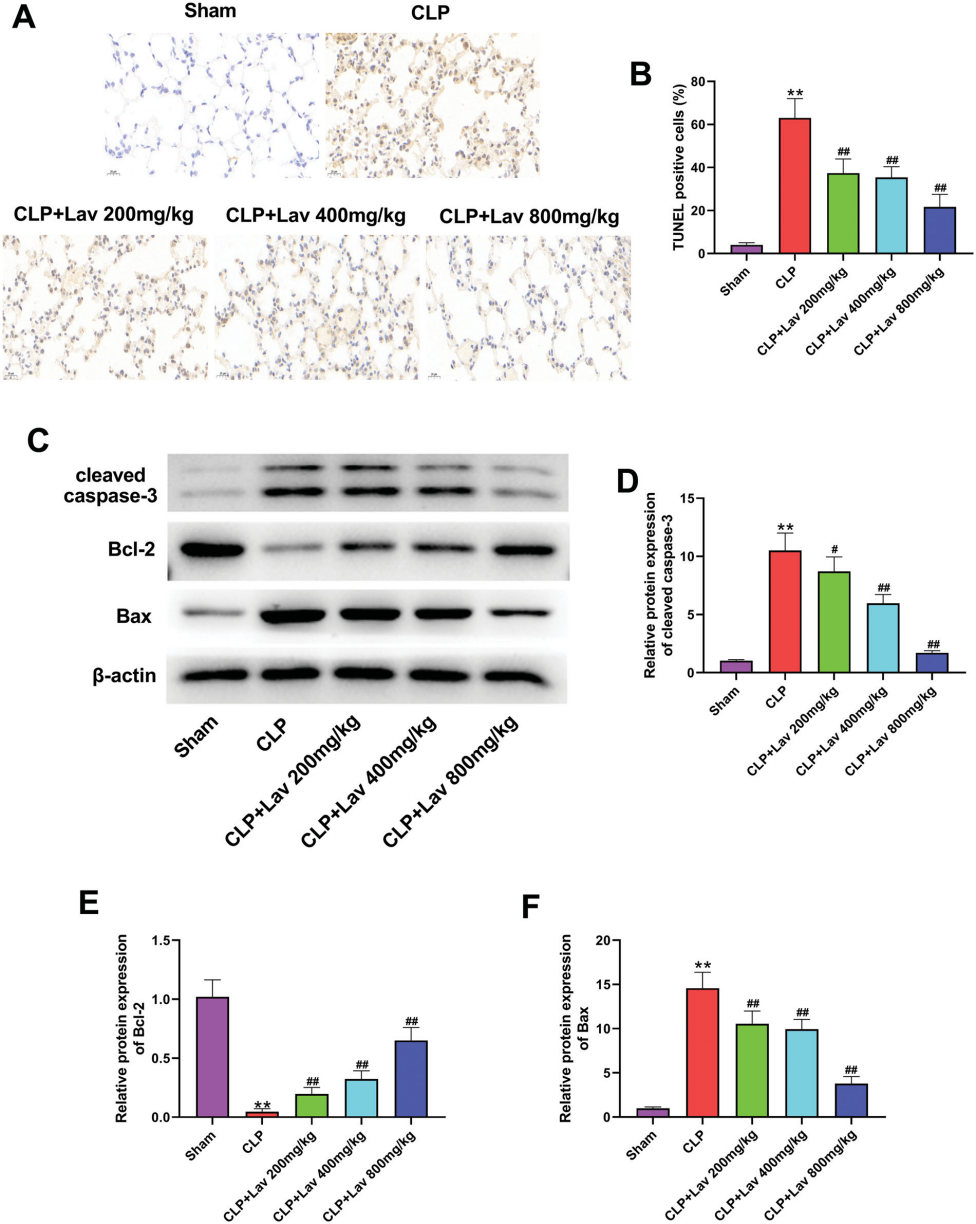
Lav pre-treatment protected the lung tissues from sepsis-induced cell apoptosis. (A and B) Cell apoptosis in the lung tissues was determined via TUNEL assay. (C–F) Cell apoptosis-associated cleaved-caspase 3, Bcl-2 and Bax expression in the lung tissues was evaluated via western blot. *Represents comparation with the Sham group, while # represents comparation with the CLP group. **p < 0.01, ^#^*p* < 0.05, ^##^*p* < 0.01.

To determine the influence of Lav pre-treatment on the apoptosis of lung tissues collected from sepsis-induced rats, a TUNEL assay and western blot were performed. The results displayed in Figures 5A and B revealed that, compared with the Sham group, the ratio of TUNEL-positive cells was dramatically augmented by CLP (*P* < 0.01), which was then declined with Lav pre-treatment (*P* < 0.01). These results were subsequently verified by variations in the levels of apoptosis-associated cleaved-caspase 3, Bcl-2 and Bax. Western blot results showed that both cleaved-caspase 3 and Bax levels were markedly elevated, except that Bcl-2 was notably reduced by CLP in comparison with the Sham group (all *P* < 0.01). Lav pre-treatment distinctly reversed these effects (Figure 5C–F, *p* < 0.05 or *p* < 0.01). The combination of these results confirmed that Lav's pre-treatment remarkably alleviated sepsis-induced apoptosis in rat lung tissues.

### Lav pre-treatment performed its protective effects on sepsis-induced ALI via deactivation of NF-κB pathway

**Figure 6.**
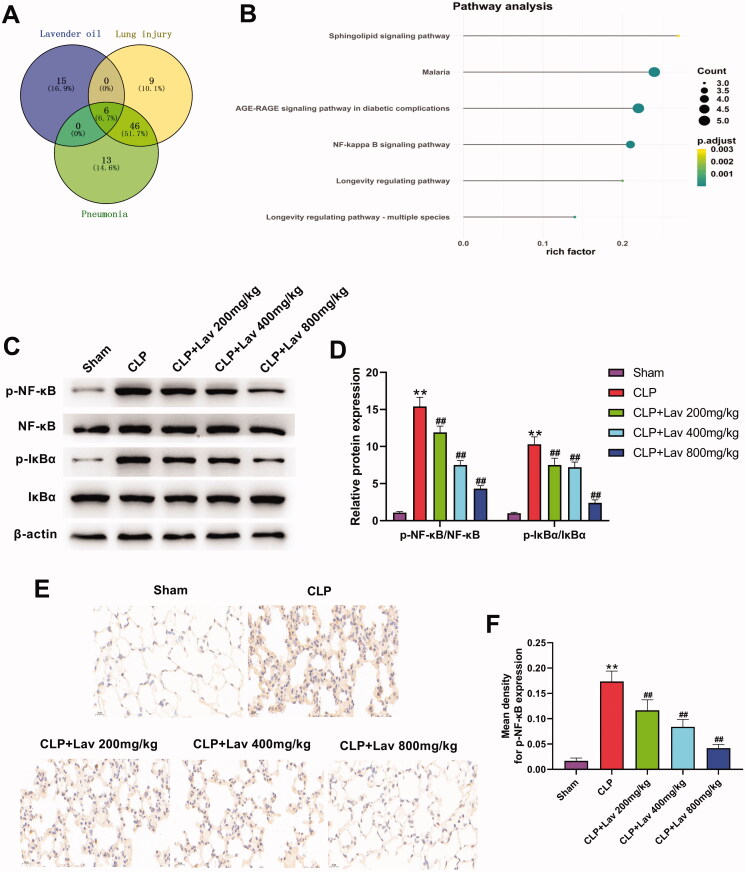
Lav pre-treatment performed its protective effects on sepsis-induced ALI via deactivation of NF-κB pathway. (A and B) Venn diagram analysis and pathway analysis were carried out to determine the potential pathway that participated in the regulation of Lav in sepsis-induced lung injury. (C and D) The levels of NF-κB pathway-associated p-NF-κB, NF-κB, p-IκBα and IκBα were measured via western blot. (E and F) Immunohistochemistry was utilised for the evaluation of the level of p-NF-κB in the lung tissues. *Represents comparation with the Sham group, while # represents comparation with the CLP group. **p < 0.01, ^##^*p* < 0.01.

To elucidate the potential regulatory mechanisms of Lav pre-treatment which exert the protective effects on sepsis-induced ALI, we explored the signalling pathways that might participate in this regulation via bioinformatic analysis. Venn diagram analysis screened out 6 overlapping signalling pathways involved in the regulation of Lav, lung injury and pneumonia (Figure 6A), which are displayed in Figure 6B. Considering that the NF-κB signalling pathway was the most widely investigated among the six pathways in lung injury, and it is the only pathway that has been confirmed as being associated with inflammation among the listed pathways, we deduced that it might also participate in the regulation of Lav in sepsis-induced ALI (de Souza Basso et al. 2021; Lee et al. 2021; Liang et al. 2021; Zhou et al. 2021). To address this assumption, we determined the expression of NF-κB-related factors by western blot and IHC. The results showed that compared with the Sham group, the ratios of p-NF-κB/NF-κB and p-IκBα/IκBα were distinctly augmented by CLP (both *p* < 0.01), which were then notably reduced by Lav pre-treatment (Figure 6C and D, all *p* < 0.01). Besides, the variations in the expression of p-NF-κB in IHC were consistent with the results in western blot (Figure 6E and F, all *p* < 0.01).

## Discussion

This research primarily investigated the efficacy of Lav pre-treatment on ALI by constructing a sepsis-induced animal model in rats. An animal model was successfully constructed in rats by performing CLP. We found that the levels of cytokines, nitrosative stress, oxidative stress and cell apoptosis in lung tissues were all distinctly elevated or enhanced by CLP. Additionally, the NF-κB pathway was markedly activated by CLP, indicating that this pathway may participate in the regulation of Lav in sepsis-induced ALI. Conversely, these CLP-induced effects were notably alleviated by Lav pre-treatment. A combination of these results indicated that Lav pre-treatment might have protective effects against sepsis-induced ALI in rats via deactivation of the NF-κB pathway.

ALI is closely correlated with various inflammatory mediators (Rajasekaran et al. 2016). Numerous studies have reported crucial roles of inflammatory cytokine networks (such as TNF-α, IL-1β and IL-6) in the amplification, mediation and perpetuation of lung injury (Matthay et al. 2012; Zhang et al. 2013; Hirano 2021). In this study, we found that CLP surgery notably elevated the expression of TNF-α, IL-1β and IL-6 in serum and BALF, which was markedly alleviated by Lav pre-treatment. These results could be consistently explained by the variations in neutrophils displayed in Figure 1F, considering that high levels of TNF-α and ILs were produced by neutrophils in a previous report (Fuller et al. 2015), and high level of TNF-α was also verified to be closely correlated with neutrophil accumulation (Presicce et al. 2020). Additionally, the inflammatory responses in sepsis/LPS-induced ALI rats were distinctly enhanced by elevating the levels of the inflammatory factors IL-6, IL-1β and TNF-α (Ju et al. 2019; Jia et al. 2021). Thus, lavender essential oil (LEO) pre-treatment effectively reduced croton oil-induced inflammatory responses in mice (Silva et al. 2015). Other studies have shown that Lav administration relieves renal ischemia/reperfusion (IR) or myocardial infarction (MI) injury in rats by reducing the levels of TNF-α and IL-1β to repress inflammation (Aboutaleb et al. 2019; Souri et al. 2019). Based on this evidence, it can be deduced that Lav pre-treatment exerts anti-inflammatory effects on sepsis-induced ALI by reducing the levels of inflammatory cytokines.

In addition to inflammation, ALI is also characterised by excessive uncontrolled apoptosis and oxidative stress (Ju et al. 2018; Zhang et al. 2019). For instance, it was reported that significant increases in cell apoptosis and oxidative stress were observed in the lung tissues of CLP-induced septic rats, which was evidenced by the augmented MPO activity and MDA content, and reduced GSH and SOD contents (Zhang et al. 2019; Jia et al. 2021). Meanwhile, a number of studies have reported the antiapoptotic and antioxidant activities of Lav in various diseases. It has been demonstrated that Lav exerts neuroprotective effects on scopolamine-induced dementia through repressing oxidative stress and apoptosis by augmenting SOD activity, reducing GSH and MDA content, and diminishing DNA cleavage patterns (Hancianu et al. 2013). Other studies have reported the cardioprotective and renal protective effects of Lav in rat models of MI and I/R injury, which were achieved by lowering the number of TUNEL-positive cells and MDA content, and elevating SOD activity (Aboutaleb et al. 2019; Souri et al. 2019). In the current study, we discovered that Lav pre-treatment markedly restrained oxidative stress in sepsis-induced rats by reducing MPO activity and MDA content, and elevating SOD and GSH contents. Furthermore, cell apoptosis was repressed by Lav pre-treatment which was confirmed by the variations in apoptosis-related cleaved-caspase 3, Bcl-2 and Bax. These results are in accordance with the literature. A combination of these findings showed that Lav pre-treatment exerted antioxidant and antiapoptotic effects on sepsis-induced ALI by increasing antioxidant enzyme production, decreasing lipid peroxidation and regulating the expression of apoptosis-related factors.

Previous studies have demonstrated the involvement of the NF-κB pathway in the regulation of inflammation and oxidative stress in lung injury (Yang et al. 2018; Wang et al. 2021). For instance, Han et al. (2018) revealed that Linarin repressed inflammation and oxidative stress in LPS-induced rats via suppression of the NF-κB and TXNIP/NLRP3 pathways. An earlier investigation discovered that activation of the NF-κB pathway promotes the initiation of the inflammatory cascade and increases the production of pro-inflammatory cytokines, which in turn facilitates inflammatory responses and neutrophil accumulation in the lungs (Kuo et al. 2011). In this study, we found that the NF-κB pathway was notably activated by CLP surgery, whereas this effect was notably reduced by Lav pre-treatment. Combined with the variations in neutrophil numbers, inflammatory cytokines and oxidative stress-related factor expression mentioned above, we conclude that Lav pre-treatment might exert its anti-inflammatory and antioxidant properties via deactivation of the NF-κB pathway.

Collectively, this study demonstrated that Lav pre-treatment might protect rats from sepsis-induced ALI by deactivating the NF-κB pathway. These findings indicate that Lav might be a candidate for the treatment of ALI in the future.

## Data Availability

The datasets used and analysed during the current study are available from the corresponding author on reasonable request.
